# Impact of online, video-based wellness training on Girl Scout leaders' wellness promotion self-efficacy, intention, and knowledge: A pilot randomized controlled trial

**DOI:** 10.3934/publichealth.2018.3.225

**Published:** 2018-07-06

**Authors:** Brooke J. Cull, Sara K. Rosenkranz, Richard R. Rosenkranz

**Affiliations:** 1 Department of Food, Nutrition, Dietetics and Health, Kansas State University, Manhattan, Kansas, 212 Justin Hall, 1324 Lovers Lane, KS 66506, United States; 2 Physical Activity and Nutrition Clinical Research Consortium, 1105 Sunset Avenue, 3rd Floor, Kansas State University, Manhattan, Kansas 66506, United States

**Keywords:** wellness promotion, nutrition, physical activity, wellness training

## Abstract

*Objective:* To examine the effectiveness of tailored, online, video-based training on Girl Scout troop leaders' wellness promotion self-efficacy, intention, and knowledge regarding physical activity and fruit and vegetable practices during troop meetings. *Design:* Randomized controlled trial. *Methods:* Thirty Girl Scouts leaders were randomized to control (CON; *n* = 16) or intervention (INT; *n* = 14) conditions. INT leaders received six online weekly training videos on implementation of physical activity (PA) and fruit and vegetable (FV) practices during troop meetings. Videos addressed leader-identified improvement areas and strategies to overcome barriers. Leaders set PA and FV goals for upcoming meetings and self-monitored their progress. Questionnaires were completed at baseline and post-intervention on leaders' task and barrier self-efficacy, intention, and knowledge regarding meeting-time PA and FV practices. *Results:* INT leaders increased PA practices barrier self-efficacy (INT = 16.5 ± 24.1%, CON = −4.8 ± 21.5%; *p* = 0.036), vegetable practices self-efficacy (INT = 10.3 ± 13.3%, CON = −3.5 ± 28.9%; *p* = 0.049), and FV practices barrier self-efficacy (INT = 12.4 ± 4.6%, CON = 1.6 ± 28.7%; *p* = 0.036), when compared to CON leaders. There were no changes (*p* > 0.05) in PA or FV knowledge. *Conclusions and implications:* Results suggest the potential for using online video-based training for improvements in wellness-promoting practices of Girl Scout troop leaders.

## 1. Introduction

Most children do not regularly engage in recommended levels of wellness-promoting behaviors, such as participating in physical activity (PA) and consuming adequate amounts of fruits and vegetables (FV). It has been estimated that only 26% of boys and 17% of girls, aged 6–19y, meet PA recommendations [1], which specify at least 60 minutes of moderate-to-vigorous PA per day [2], and rates of activity decline with age [3]. Additionally, 60% of children do not consume the recommended amount of fruits, and 93% do not consume an adequate amount of vegetables [4]. Both PA behaviors and FV consumption patterns established in childhood are shown to carry into adulthood [5],[6], so intervening in childhood to target development of healthful behaviors represents an important research area.

Interventions meant to improve healthful behaviors should be delivered in settings where children spend a majority of their time, including homes, schools, and community-based settings [7], and research suggests that interventions delivered in these settings have the potential to positively impact children. One community-based setting that has been studied for wellness promotion is Girl Scouts (GS) of America. Reaching more than 2.5 million girls and adult volunteers (GirlScouts.org), this organization is a viable channel for impact on a large, diverse group of girls. GS leaders can offer opportunities for PA and healthy snacking during their meetings, and they may choose to work toward badges and awards that include wellness-promoting components. Previous research has shown positive outcomes in wellness practice implementation during troop meetings following leader-targeted interventions, specifically in the areas of PA opportunities and healthy snacking.

A previous GS intervention included an educational curriculum delivered by leaders and the implementation of wellness-promoting policies during troop meetings [8]. That intervention increased PA levels of girls and improved the availability of FV offered as snack choices during the meeting. A more recent study compared the effectiveness of two GS leader wellness-training delivery methods, in-person versus online [9]. Results showed that in-person training was more effective than online training for implementation of wellness-promoting opportunities during troop meetings. However, authors concluded that there was a need for further research evaluating online delivery methods, as this may represent a more cost-effective and further-reaching platform for dissemination and implementation of wellness-promoting practices in GS.

One type of delivery method that may be feasible and effective for use in GS is online video-based training. Online video-based leader wellness training may offer a novel, sustainable, cost-effective, and scalable way to help maximize the reach and dissemination of training for implementation of wellness-promoting opportunities within the GS organization. When compared to a standard website, video messages delivered online may be tailored to each troop leader and their specific needs, and thus may increase the acceptability and effectiveness of this training method.

The purpose of this pilot study was to evaluate the effectiveness of six weeks of tailored, online wellness-training videos on determinants of wellness-promotion practices in GS leaders. We hypothesized that leaders in the intervention condition would increase self-efficacy, intention, and knowledge from baseline to post-intervention, when compared to the control group.

## 2. Methods

### 2.1. Participants and recruitment

Participants for this pilot randomized controlled trial were thirty GS troop leaders from seven counties within northeast Kansas. Participants were recruited at their monthly leader meetings, where they received wellness-promotion practices implementation training, completed a questionnaire regarding their troop demographics and usual meeting practices, and indicated whether they would be interested in participating in a wellness-promotion project. Fifty-two leaders indicated interest in participation, and 30 leaders were reached via email, had Internet access, and consented to randomization and enrollment. All participants provided written consent, and this study was approved by Kansas State University's Institutional Review Board.

Leaders within each county were randomized to either the intervention or control group (1:1 allocation), with county serving as the blocking factor, using an online randomization program.

### 2.2. Intervention

Participants in the control group (*n* = 16) received usual care, in that they had access and were directed to existing, publicly available, wellness-promotion resources, while the intervention group (*n* = 14) received weekly, tailored training videos over the course of six weeks. These videos were uploaded to a video-streaming website, and leaders received an email with their specific video link each week. Each video focused on implementation of either PA or healthy snacking (offering FV) during the troop meeting, tailored to the leader based on their usual meeting practices and leader-identified barriers. Each 3–5 minute video featured a research assistant reading a tailored script. Various theoretically informed behavior change techniques were included in each video, and the videos were meant to build upon one another. The training video content and evidence-based techniques for behavior change are described in Table 1. Videos were created individually for each leader, and addressed specific concerns that leaders had identified as barriers during their baseline assessment.

**Table 1.** Wellness-training video content and associated behavior change techniques.

Training Video Content	Behavior Change Techniques
Recommendations for health behavior identified	Provide information about behavior in general Provide normative information about others' behavior
Benefits of incorporating wellness practice	Provide information about the behavior specific to the individual
Discussion of leader-specified barrier to wellness practice, and solutions for overcoming it	Barrier identification/problem-solving
Links to videos and pictures depicting other troops' activities and ideas to overcome the same barrier	Provide instruction on performing behavior Model/demonstrate behavior
Reflection on troop's usual practice and where improvement can be made	Review of behavioral goals Feedback on performance
Specific goal setting for upcoming troop meeting	Behavioral goal setting Action planning Set graded tasks Time management
Encouragement for troop leader	Rewards contingent on effort or progress toward behavior Social support

### 2.3. Measures

Informed by Social Cognitive Theory and Theory of Planned Behavior, outcome measures were created for the leaders' behavior-related psychosocial factors of self-efficacy and intention for offering PA and FV consumption opportunities, as well as leaders' knowledge regarding PA and FV consumption in children. Both task self-efficacy (confidence to offer specific, distinct levels of PA and FV during the meeting) and barrier self-efficacy (confidence to overcome specific barriers to offering PA and FV during the meeting) were assessed. The same questionnaires were completed at baseline and post-intervention. The questionnaires, specific scenarios to consider, and scoring procedures are available as Supplement 1. Leaders completed these self-report measures via an online questionnaire housed in the university's online learning management system.

The self-efficacy measures were created using Bandura's “Guide for Constructing Self-Efficacy Scales” [10]. For all self-efficacy questionnaires, leaders reported their level of confidence for offering specific opportunities for their girls during the troop meeting, in 10-point increments, on a scale of 0 (cannot do at all) to 100 (highly certain can do). A total mean self-efficacy score was computed for each measure. Internal consistency for each measure was high (Cronbach's α > 0.84).

Intention for offering PA opportunities, fruits, and vegetables, were each assessed via two items using a 5-point Likert scale, modified from items used in previous work [11]. For the first item, leaders were asked whether they intended to offer opportunities at upcoming troop meetings, and the second asked whether they had a plan to offer opportunities. Cronbach's α values were 0.97 (PA), 0.98 (fruit), and 0.97 (vegetable).

Knowledge regarding PA and FV consumption in children was assessed via four items each. Leaders were asked about knowledge of recommendations, the percentage of children meeting recommendations, and benefits of PA and FV. Each item was scored as incorrect (0 points) or correct (1 point), for a maximum score of four on each measure.

### 2.4. Statistical analysis

Data analyses were performed using SPSS statistical software (Version 23.0, IBM SPSS). Troop and leader demographics were compared at baseline using Chi-squared test. Summary scores from the questionnaire were calculated, where appropriate. Repeated measures ANOVA was used to assess change over time between groups on outcome measures where parametric assumptions were met, including PA self-efficacy and PA barrier self-efficacy. Non-parametric alternatives were used in cases where parametric assumptions were not met. For all tests, significance was set at *p* < 0.05.

## 3. Results

Baseline troop characteristics are shown in Table 2. There were no differences (*p*s > 0.05) between groups at baseline for the studied characteristics.

**Table 2.** Baseline troop characteristics.

	Control (*n* = 16)	Intervention (*n* = 14)
Troop Level		
Daisy (K–1^st^ Grade)	2 (12.5%)	0 (0%)
Brownie (2^nd^–3^rd^ Grade)	9 (56.3%)	5 (35.7%)
Junior (4^th^–5^th^ Grade)	3 (18.8%)	5 (35.7%)
Cadette (6^th^–8^th^ Grade)	2 (12.5%)	3 (21.4%)
Leader SES		
Low-income	8 (43.8%)	6 (42.9%)
Not low-income	7 (50.0%)	6 (42.9%)
Unreported	1 (6.3%)	2 (14.3%)
Fruit Availability During Meetings		
Rarely or Never	4 (25%)	5 (35.7%)
At Least Sometimes	12 (75%)	9 (64.3%)
Vegetable Availability During Meetings		
Rarely or Never	11 (68.8%)	5 (35.7%)
At Least Sometimes	5 (31.3%)	9 (64.3%)
Physical Activity Opportunity During Meetings		
Rarely or Never	2 (12.5%)	1 (7.1%)
At Least Sometimes	14 (87.5%)	13 (92.9%)

### 3.1. PA practices self-efficacy

At baseline, the highest mean self-efficacy was for offering at least a few minutes of PA (96.3 ± 9.3% confidence), and the lowest was for offering at least 30 minutes of PA (34.3 ± 31.5% confidence). There were no differences, between or within groups, for changes in leader-reported mean self-efficacy for offering PA opportunities for girls during troop meetings (INT = 7.6 ± 17.9%, CON = 2.2 ± 18.1%; *p* = 0.48). Mean self-efficacy, by group and time, are shown in Table 3.

**Table 3.** Self-efficacy by condition and time.

Self-Efficacy Category	CON Baseline	CON Post	INT Baseline	INT Post
PA Practices	65.4 ± 18.0	70.2 ± 15.9	69.6 ± 21.9	74.6 ± 21.1
PA Barriers†	73.8 ± 19.0	68.1 ± 20.9	69.3 ± 18.4	83.6 ± 13.2
Fruit Practices	64.1 ± 28.3	57.5 ± 26.8	60.9 ± 29.9	62.0 ± 28.7
Vegetable Practices†	57.8 ± 31.0	52.3 ± 31.6	54.1 ± 27.7	59.0 ± 29.7
FV Barriers†	67.7 ± 27.8	68.7 ± 18.1	60.8 ± 23.4	72.4 ± 29.8

Note: †Significant condition x time interaction (*p* < 0.05). INT group had greater increases in self-efficacy related to PA barriers, vegetable practices, and FV barriers.

### 3.2. PA practices barrier self-efficacy

There was a significant group-by-time interaction for PA practices barrier self-efficacy, whereby leaders in the intervention group reported higher self-efficacy to overcome PA barriers at post-intervention, when compared to control (INT = 16.5 ± 24.1%, CON = −4.8 ± 21.5%; *p* = 0.04). Intervention leaders reported greater improvements in self-efficacy to overcome barriers related to shortage of time (*p* = 0.02) and when the girls seemed too tired for PA (*p* = 0.03).

### 3.3. Fruit practices self-efficacy

There were no differences, between or within groups, for changes in leader-reported mean self-efficacy for offering fruit as a snack choice (INT = 7.3 ± 11.0%, CON = −4.6 ± 28.0%; *p* = 0.12).

### 3.4. Vegetable practices self-efficacy

In the intervention group, leader self-efficacy for offering vegetables as a snack increased (INT = 10.3 ± 13.3%, CON = −3.5 ± 28.9%; *p* = 0.049). Intervention leaders reported a greater increase in self-efficacy for offering at least one serving of vegetable at all meetings, when compared to control leaders (*p* = 0.04).

### 3.5. FV practices barrier self-efficacy

Intervention leaders reported a greater increase in self-efficacy to overcome barriers associated with offering FV as snack choices during the troop meeting, when compared to the control group (INT = 12.4 ± 4.6%, CON = 1.6 ± 28.7%; *p* = 0.04). Intervention leaders reported increased self-efficacy for overcoming the barriers related to limited money (*p* = 0.03) and limited time for preparation (*p* = 0.04).

### 3.6. PA and FV intention

At baseline, 100% of leaders reported that they “agree” or “strongly agree” that they were intending to offer enough PA to meet troop goals during upcoming meetings, with no changes over time.

**Figure 1.**
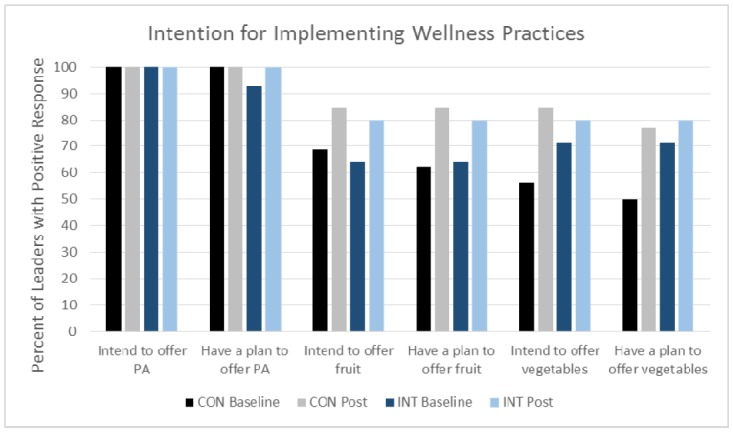
Percentage of leaders with positive PA and FV intentions.

At baseline, the majority of troop leaders reported that they intended to offer fruits and vegetables as snack choices. There was a significant increase in intention for offering fruits (*p* = 0.02) and vegetables (*p* = 0.02) during upcoming meetings, with no difference between groups. Intention for implementing wellness practices is shown in Figure 1.

Figure 1 caption: Intention for offering fruits (*p* = 0.02) and vegetables (*p* = 0.02) increased from baseline to post-intervention, with no differences between groups.

### 3.7. PA and FV knowledge

There were no changes, between or within groups, for PA knowledge (INT = 0.30 ± 1.06%, CON = −0.38 ± 1.19%; *p* = 0.61) nor FV knowledge (INT = 0.10 ± 0.57%, CON = 0.0 ± 0.81%, *p* = 1.00).

## 4. Discussion

The purpose of this pilot study was to evaluate the effectiveness of six weeks of tailored, online wellness-training videos on determinants of wellness-promotion practices in GS leaders. We hypothesized that leaders in the intervention group would increase their self-efficacy, intention, and knowledge, and our hypotheses were partially supported. When compared to the control group, intervention leaders increased their PA practices barrier self-efficacy, vegetable practices self-efficacy, and FV practices barrier self-efficacy. Both groups increased their intention for offering fruits and vegetables during upcoming meetings. However, there were no changes in PA or FV knowledge following the video training intervention.

Self-efficacy is often positively associated with health behavior change, as well as offering healthier opportunities for children. This construct relates to beliefs about capabilities for successfully functioning in an event or situation [12]. A meta-analysis indicated that interventions found to be most successful at increasing self-efficacy included elements of action planning, instruction, and reinforcement toward the desired behavior, as well as self-monitoring, feedback, and setting behavioral goals [13]. Evidence shows that caregiver self-efficacy to promote healthy behaviors is associated with child sedentary time, water consumption, and FV intake [14], thus showing that increasing self-efficacy of those in a position to offer healthy opportunities for children is important. Our wellness-training videos employed techniques to increase leaders' self-efficacy for offering PA and FV consumption opportunities, as well as overcoming associated barriers during troop meetings, and it appears that the video training intervention was effective for eliciting improvements in these areas.

In the Theory of Planned Behavior, intention is the most proximal determinant of behavior, and has been shown to predict approximately 30% of the variance of future action [15]. However, there is an established gap between intention and behavior, and a meta-analysis showed a positive association between intention and behavior across a range of activities, with a correlation of only 0.53 [16]. Although increases in intention may not fully translate into a behavior change for all leaders, it is encouraging that the current study showed improvements in this area for increases in FV consumption opportunities during troop meetings for both groups.

There were no changes in knowledge over the course of the intervention, possibly due to a ceiling effect, where the leaders already scored relatively high on the knowledge questionnaire at baseline. As such, we would be less likely to see significant improvements in their scores. Knowledge of standards or target behaviors is important for health behavior change, but research does not consistently support the idea that increasing knowledge leads to improvements in health behaviors [17]. However, evidence has shown that adults' knowledge of recommendations or standards is associated with being more supportive of PA for children [18].

Our wellness-training videos were specifically tailored to each troop leader. Tailored communication is defined as “any combination of strategies and information intended to reach one specific person, with communication uniquely individualized to that person, related to the outcome of interest, and derived from an individual assessment” [19]. Previous evidence suggests that tailored messages appear more relevant to the user [20],[21]. A meta-analysis found that tailored messages were more effective for eliciting health behavior change than generic forms of communication [22]. Additionally, interventions featuring more than one contact with participants were more effective than a single, tailored message. Interventions that included tailoring based on theoretical concepts, including self-efficacy and social support, were more effective than those not based on theory [22]. Our tailored, online wellness-training videos incorporated these components shown to be important for health behavior change.

A strength of our study was that we recruited and studied participants across seven counties, including both rural and urban troops. Since the training videos were uploaded to a video-streaming website, leaders could access the content from anywhere. Streaming capability may increase the potential for widespread dissemination, including reaching those leaders who may be in isolated or otherwise hard-to-reach areas. Being a pilot study, there were some limitations as well. The primary outcomes of this study relied on leader self-report, and it is possible that responses do not accurately reflect true characteristics. Individually tailoring video messages for each troop leader required considerable time, and may not be scalable for a larger population. However, future research may study the effectiveness of tailoring messages to specific group needs, rather than each individual. Additionally, future research should determine whether or not changes actually occur in wellness promotion practices within troop meetings.

There is a need to develop and evaluate the effectiveness of interventions and tailored training methods capable of reaching those hard-to-reach leaders in important settings. Online, video-based training has been shown to be effective for impacting the determinants of wellness behaviors, potentially leading to more wellness-promoting environments for children. If implemented across the organization and other similar organizations, it is possible for online, video-based wellness-training to impact healthful behaviors of children.
