# Supplementary material for: Impact of online, video-based wellness training on Girl Scout leaders' wellness promotion self-efficacy, intention, and knowledge: A pilot randomized controlled trial
Source: AIMS Public Health. 2018 Jul 6;5(3):225–34. doi: 10.3934/publichealth.2018.3.225 (PMC6141553; doi:10.3934/publichealth.2018.3.225)
Supplement: Supplementary file 1 [file publichealth-05-03-225-s01.pdf]

Public Health

http://www.aimspress.com/journal/aimsph

AIMS Public Health, 5 (3): 225–234.
DOI:
10.3934/publichealth.2018.3.225
Received: 25 April 2018
Accepted: 11 June 2018
Published: 04 July 2018

Research article

Impact of online, video-based wellness training on Girl Scout leaders' wellness promotion self-efficacy, intention, and knowledge: A pilot randomized controlled trial

Brooke J. Cull[1,2,*], Sara K. Rosenkranz[1,2] and Richard R. Rosenkranz[1,2]

[1] Department of Food, Nutrition, Dietetics and Health, Kansas State University, Manhattan, Kansas, 212 Justin Hall, 1324 Lovers Lane, KS 66506, United States
[2] Physical Activity and Nutrition Clinical Research Consortium, 1105 Sunset Avenue, 3rd Floor, Kansas State University, Manhattan, Kansas 66506, United States

* **Correspondence:** Email: brooke1@ksu.edu; Tel: +7855320170.

Supplemental File 1. Self-Efficacy, Intention, and Knowledge Questionnaire

Practice Rating

To familiarize yourself with the rating form, please complete this practice item first. If you were asked to lift objects of different weights **right now,** how certain are you that you can life each of the weights described below?

Rate your degree of confidence by recording a number from 0 to 100 using the scale given below:

0	10	20	30	40	50	60	70	80	90	100
Cannot do at all					Moderately can do				Highly certain can do	

Confidence (0-100)

Physical Strength

Lift a 10 pound object	________________________
Lift a 50 pound object	________________________
Lift a 100 pound object	________________________
Lift a 200 pound object	________________________
Lift a 300 pound object	________________________

Physical Activity Confidence

Please rate how certain you are that you can offer the following physical activity opportunities during a typical upcoming troop meeting (excluding special events and celebrations).

Rate your level of confidence by recording a number from 0 to 100 using the scale given below:

0	10	20	30	40	50	60	70	80	90	100
Cannot do at all					Moderately can do				Highly certain can do	

	Confidence (0-100)
Offer at least a few minutes of physical activity	________________________
Offer at least 10 minutes of physical activity	________________________
Offer at least 15 minutes of physical activity	________________________
Offer at least 20 minutes of physical activity	________________________
Offer 30 minutes or more of physical activity	________________________

Fruit and Vegetable Availability Confidence

Please rate how certain you are that you can offer the following fruit and vegetable opportunities for snack time during a typical upcoming troop meeting (excluding special events and celebrations).

Rate your level of confidence by recording a number from 0 to 100 using the scale given below:

0	10	20	30	40	50	60	70	80	90	100
Cannot do at all					Moderately can do				Highly certain can do	

	Confidence (0-100)
Offer at least 1 serving of **fruit** at some meetings (greater than 0% of meetings, but less than 50% of meetings)	________________
Offer at least 1 serving of **fruit** at least every other meeting (50% or more of meetings)	________________
Offer at least 1 serving of **fruit** at ALL meetings (100% of meetings)	________________
Offer 2 or more servings of **fruit** at ALL meetings (100% of meetings)	________________
Offer at least 1 serving of **vegetables** at some meetings (greater than 0% of meetings, but less than 50% of meetings)	________________
Offer at least 1 serving of **vegetables** at least every other meeting (50% or more of meetings)	________________
Offer at least 1 serving of **vegetables** at ALL meeting (100% of meetings)	________________
Offer 2 or more servings of **vegetables** at ALL meetings (100% of meetings)	________________

Confidence with Barriers to Physical Activity Time

A number of situations are described below that can make it hard to offer physical activity opportunities during your troop meetings. For each blank, please rate how certain you are that you can offer physical activity opportunities for your girls during a troop meeting when faced with the following challenges.

Rate your level of confidence by recording a number from 0 to 100 using the scale given below:

0	10	20	30	40	50	60	70	80	90	100
Cannot do at all					Moderately can do				Highly certain can do	

	Confidence (0-100)
**Example: When we have visitors**	**<u>100 (highly certain can do)</u>**
When you are running short on time	________________
When you have a lot of other activities planned	________________
When you don't have a lot of room	________________
During bad weather	________________
When the girls seem tired	________________
When you haven't planned out a physical activity	________________
When you can't think of any activities to do	________________
When you don't have any physical activity equipment	________________

If you have any comments regarding barriers to physical activity, or if you have additional ones that weren't listed, please tell us below.

____

____

Confidence with Barriers to Fruit and Vegetable Availability

A number of situations are described below that can make it hard to offer fruits and vegetables as snack choices during your troop meetings. In each blank, please rate how certain you are that you can offer fruit and vegetable opportunities for your girls during a troop meeting when faced with the following challenges.

Rate your level of confidence by recording a number from 0 to 100 using the scale given below:

0	10	20	30	40	50	60	70	80	90	100
Cannot do at all					Moderately can do				Highly certain can do	

	Confidence (0-100)
When you don't have a lot of money to spend	________________
When you don't have a lot of time to prepare the snack	________________
If the girls are picky about the snack options	________________
If parents typically bring snacks	________________
If you don't have kitchen facilities	________________
If you can't think of any fruit or vegetable snack ideas	________________
If you don't have any kitchen supplies to prepare the snack	________________

If you have any comments regarding barriers to providing fruits and vegetables, or if you have additional ones that weren't listed, please tell us below.

____
____

____
____

Physical Activity and Snacking Intention Survey

On the following items, please indicate your level of agreement with each statement about offering physical activity and fruits and vegetables during a typical upcoming troop meeting (excluding special events or celebrations) on the 1 to 5 scale.

1=strongly disagree; 2= disagree; 3=neither agree or disagree; 4= agree 5=strongly agree

I intend to offer enough **physical activity** to meet our troop goal during upcoming troop meetings

1 2 3 4 5

I am planning to offer enough **physical activity** to meet our troop goal during upcoming troop meetings

1 2 3 4 5

I intend to offer enough **fruits** to meet our troop goal during upcoming troop meetings

1 2 3 4 5

I am planning to offer enough **fruits** to meet our troop goal during upcoming troop meetings

1 2 3 4 5

I intend to offer enough **vegetables** to meet our troop goal during upcoming troop meetings

1 2 3 4 5

I am planning to offer enough **vegetables** to meet our troop goal during upcoming troop meetings

1 2 3 4 5

Physical Activity and Fruit and Vegetable Knowledge Survey

For each of the following questions, please mark your response regarding physical activity and fruit and vegetable consumption for girls.

How many minutes of physical activity should kids get every day?

A. 15 minutes
B. 30 minutes
C. 45 minutes
D. At least 60 minutes

What percentage of girls meet the recommendations for physical activity?

A. Less than 20%
B. 25-40%
C. 45-60%
D. Greater than 75%

Girls are typically more physically active than boys

A. True
B. False

Which of these is/are a potential benefit of being physically active (circle all correct answers)?

A. Better school performance
B. Weight control
C. Decreased risk of chronic disease
D. Improved mood

What is the recommendation for how many combined servings of fruits and vegetables kids should eat every day?

A. 2 servings
B. 3 servings
C. 4 servings
D. 5 or more servings

Approximately what percentage of youth meet the recommendations for fruit consumption?

A. Less than 20%
B. 40%
C. 60%
D. 80%

Approximately what percentage of youth meet the recommendations for vegetable consumption?

A. Less than 20%
B. 40%
C. 60%
D. 80%

Which of these is/are a benefit of consuming fruits and vegetables (circle all correct answers)?

A. Decreased risk of chronic disease
B. Contain fiber, vitamins, and minerals
C. Low in calories
D. Quick, natural snack choice

AIMS Press

© 2018 the Author(s), licensee AIMS Press. This is an open access article distributed under the terms of the Creative Commons Attribution License (http://creativecommons.org/licenses/by/4.0)
